# Effect of propolis extract addition on some physicochemical, microbiological, and sensory properties of kefir drinks

**DOI:** 10.1002/fsn3.3671

**Published:** 2023-09-07

**Authors:** Sinem Bengi, Oguz Gursoy, Hande Özge Güler Dal, Yusuf Yilmaz

**Affiliations:** ^1^ Division of Food Engineering, Graduate School of Natural and Applied Sciences Burdur Mehmet Akif Ersoy University, Istiklal Campus Burdur Turkey; ^2^ Department of Food Engineering, Faculty of Engineering and Architecture Burdur Mehmet Akif Ersoy University, Istiklal Campus Burdur Turkey

**Keywords:** antioxidant, functional food, health, kefir, propolis

## Abstract

Kefir drinks with sugar (5%, w/v), strawberry flavor (0.15%, v/v), and propolis extract (PE) at different ratios (0.150%, 0.225%, and 0.300%, v/v) were produced and stored at 4°C, and their physicochemical, rheological, microbiological, and sensory properties were monitored during storage. The ratio of PE and storage time had an insignificant effect on the dry matter, protein, fat contents, Commission Internationale de l'Eclairage (CIE) L* and a* color values, apparent viscosity, consistency coefficient, flow behavior index, *Lactobacillus* spp., *Lactococcus* spp., and yeast counts of kefir drinks (*p* > .05). The CIE b* values of kefir drinks increased with an increase in PE ratio (*p* < .05). All kefir samples exhibited a pseudoplastic flow behavior. Initially, the total antioxidant capacity of kefir drinks was 2.19 μmol TE/100 mL, which increased to 2.51 μmol TE/100 mL for kefir drinks with 0.225% PE. The total phenolic content and antioxidant capacity of kefir drinks with PE decreased during storage. PE addition did not influence the sensory color and taste liking scores of kefir drinks adversely until the 8th day of storage. In terms of odor liking scores, kefir drinks with 0.225% and 0.300% PE had a similar score to control drinks. Additionally, kefir drinks with 0.150% and 0.225% PE received an overall liking score similar to control drinks. Results indicated that the incorporation of PE at a ratio of 0.225% was recommended for the production of strawberry‐flavored kefir drinks with acceptable sensory characteristics and increased functional properties, and this product could be stored for up to 8 days.

## Graphical abstract

Kefir drinks with sugar (5%, w/v), strawberry flavor (0.15%, v/v), and propolis extract (PE) at different ratios (0.150%, 0.225%, and 0.300%, v/v) were produced and stored at 4°C, and their physicochemical, rheological, microbiological, and sensory properties were monitored during storage. Initially, the total antioxidant capacity of kefir drinks was 2.19 μmol TE/100 mL, which increased to 2.51 μmol TE/100 mL for kefir drinks with 0.225% PE. Results indicated that the incorporation of PE at a ratio of 0.225% was recommended for the production of strawberry‐flavored kefir drinks with acceptable sensory characteristics and increased functional properties, and this product could be stored for up to 8 days.

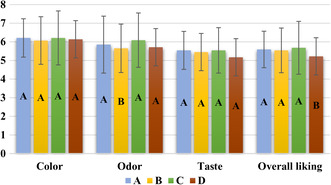

## 1 INTRODUCTION

Studies on the probiotic effects of fermented dairy products have increased in recent years, and kefir, a major dietary source of unique probiotic microorganisms, has also been the subject of these studies. Originating from the Balkans, Eastern Europe, and the Caucasus, kefir is a commercially produced fermented dairy product that is produced by kefir grains and contains natural probiotics such as *Lactobacillus acidophilus*, *Bifidobacterium bifidum*, as well as various lactic acid bacteria and yeasts with a partially foamy, thick consistency, and slightly sour taste (Koroleva, 1988; Kök‐Taş et al., 2013). Kefir grains are irregular particles with a soft, gelatinous texture, ranging in color from yellow to white, and varying in diameter between 3 and 20 mm. The composition of kefir grains primarily consists of *Lactobacillus*, *Leuconostoc*, *Acetobacter*, *Streptococcus*, and various yeasts (Garrote et al., 2001; Irigoyen et al., 2005). Kefir is a very valuable fermented drink for human nutrition because of its health beneficial effects. While it contains almost all nutrients in milk, it has an increased nutritional value for human body via various microorganisms in the structure of kefir grains (Karagözlü, 2003). The health benefits of kefir consumption can be attributed to the fact that it is a natural probiotic besides its high protein, mineral, and vitamin contents (Zourari & Anifantakis, 1988). Previous studies have constantly reported the health beneficial effects of kefir consumption such as its anticarcinogenic, antibacterial, antitumoral, antimicrobial, immunomodulatory, blood cholesterol and blood pressure, and digestive system regulatory effects (Kesenkaş et al., 2017).

In kefir production, various milk types such as cow, sheep, goat, buffalo, rice, and soy milk or a mixture of them with different contents of fat and skim milk can be used (Bensmira & Jiang, 2012; Karagözlü, 2003). Thus, the sensory and nutritional properties of kefir drinks are mostly dependent on the composition of milk, microbiological characteristics of grains and starters used, production method, storage time, and addition of different ingredients (Zourari & Anifantakis, 1988). Thermophilic and mesophilic probiotic bacteria and yeasts are mostly used together or separately as starter cultures in kefir production (Kadıoğlu, 2017; Zourari & Anifantakis, 1988). Nowadays, the use of lyophilized cultures in industrial production saves time and facilitates production while providing hygienic and standardized production (Özcan et al., 2018). In the production of fruity kefirs, various types of flavoring agents and fruit sauces can be added to plain kefir drinks.

Propolis is a natural product produced by honey bees using substances such as resin, mucilage, and gum in plant and tree leaves. It contains more phenolic components than other bee products and has strong antioxidant activity (Mehmetoğlu et al., 2017; Viuda‐Martos et al., 2008; Wang et al., 2018; Zabaiou et al., 2017; Zheng et al., 2023). Propolis, considered a natural product with medicinal properties, has been used as a supplement in the therapy of many diseases since ancient times, and PE is extremely complex in its nature because of the many chemical constituents it contains (Russo et al., 2002; Viuda‐Martos et al., 2008). Recently, studies on propolis in the literature and its use as a dietary supplement have increased steadily. In general, half of raw propolis is composed of resin and plant balm while the remaining contains bee wax (30%), essential and aromatic oils (10%), pollen (5%), and other organic substances (5%) (Burdock, 1998; Sforcin, 2007). There are more than 850 isolated compounds in the composition of propolis, which includes phenolic substances and their esters, caffeic acid phenyl ester (CAPE), flavonoids, terpenes, aromatic acids, aromatic aldehydes and alcohols, beta‐steroids, vitamins, etc. (Koru et al., 2007; Orhan et al., 1999; Šturm & Ulrih, 2020; Viuda‐Martos et al., 2008).

The high antioxidant property of propolis is derived from its polyphenols like flavonoids and phenolic acids in its composition. Rich phenolic content of propolis creates a strong antioxidant effect and may protect human health against the damage of oxidative stress (Castaldo & Capasso, 2002; Viuda‐Martos et al., 2008; Zabaiou et al., 2017).

In the literature, there are few studies on the use of propolis in the production of various milk or dairy products. Propolis was previously added to dairy products such as Ras cheese (Aly & Elewa, 2007), yogurt (Çelik, 2016; Çifci, 2015; Güney, 2016), and ice cream (Mehmetoğlu, 2019). In a preliminary study by Chon et al. (2020), propolis was used in kefir production among different dairy products. However, the effects of propolis on the microbiological and rheological properties, antioxidant activities, and phenolic contents of kefirs were not investigated in this preliminary study. The aim of this present study was to incorporate propolis extract into kefir drinks in order to develop a new functional product by obtaining a synergistic effect of these two products with proven health benefits. To facilitate the consumption of kefir drinks and increase its accessibility by different segments of population, propolis extract was added into strawberry‐flavored kefir drinks at different ratios, and the physicochemical, rheological, antioxidant, microbiological, and sensory properties of drinks were monitored during storage at 4°C.

## 2 MATERIALS AND METHODS

### 2.1 Materials

Cows' milk with standardized dry matter, fat contents, and sugar was obtained from a national market in Burdur (Turkey). Kefir culture (Vivo Gıda San. ve Tic. Ltd. Şti., Istanbul, Turkey), water‐soluble strawberry flavor (Aromsa Besin Aroma Katkı Mad. San. Tic. A.Ş., Kocaeli, Turkey), and propolis extract (BEEO/Bioactive, SBS Bilim Bio Çözümler San. ve Tic. A.Ş., Istanbul, Turkey) in water‐soluble form with 10% purity were used in the production of kefir drinks. According to the producer of the propolis extract, the dry matter content of propolis extract used in this study was 10.1%. The total phenolic content, total flavonoid content, and antioxidant capacity of the extract were 40.0 mg gallic acid equivalent (GAE)/mL, 38.0 mg catechin equivalent (CE)/mL, and 122.2 mg Trolox® equivalent (TE)/mL, respectively. According to the manufacturer, the main phenolic compounds in propolis extract determined by LC–MS/MS included caffeic acid phenethyl ester (7081.9 mg/L), chrysin (3215.6 mg/L), ferulic acid (640.9 mg/L), pinocembrin (641.3 mg/L), caffeic acid (603.9 mg/L), hesperetin (249.3 mg/L), p‐coumaric acid (226.0 mg/L), rhamnetin (225.3 mg/L), and apigenin (298.2 mg/L).

### 2.2 Methods

#### 2.2.1 Preparation of kefir drinks

Cow milk used in kefir production was initially heat treated at 90°C for 5 min and then cooled to the fermentation temperature of 30°C. Later, kefir starter culture (1 g per 5 L milk) was added to the milk, and samples were incubated at 23.5°C for about 20 h, as instructed by the manufacturer of the culture. After their pH reached 4.18 ± 0.1, maturation process was carried out under refrigeration conditions at +4 ± 1°C for 24 h. Then, kefir samples with sugar (5.00%, w/v), strawberry flavor (0.15%, v/v), and different ratios of PE (0%, 0.150%, 0.225%, and 0.300%, v/v) were produced, coded as A, B, C, and D, respectively. The concentrations of strawberry flavor and PE in kefir drinks were determined according to the instructions of their manufacturers. The daily consumption of water‐soluble PE was recommended as 0.30 mL for children and 0.60 mL for adults by its manufacturer. Therefore, 0.150 and 0.300% PE ratios were selected for the daily consumption of 200 mL kefir drinks, which could correspond to the recommended daily consumption rates of children and adults, respectively. To suppress the bitterness of PE in kefir drinks and to facilitate their consumption, table sugar and strawberry flavor were added to samples. The ratio of sugar in kefir drinks was determined by preliminary experiments and according to the amount of sugar in commercial fruit‐flavored kefir drinks currently sold in markets. Preliminary experiments revealed that kefir drinks had an unpleasant bitter taste beyond 8 days of storage at +4 ± 1°C; therefore, kefir samples were stored for a maximum of 8 days. Some physicochemical, antioxidant, rheological, microbiological, and sensory properties of kefir drinks were determined on the 1st, 4th, and 8th days of storage.

#### 2.2.2 Physicochemical analyses

Dry matter contents (%) of kefir drinks were determined by using a rapid moisture analyzer (Kern DBS 60‐3, Kern & Sohn GmbH, Balingen, Germany) at 110°C. pH values were determined by a pH meter (Jenco 6173, Jenco, San Diego, CA, USA). Acidity (% lactic acid) was determined according to the method of Metin and Öztürk (2016). The total nitrogen content of kefir samples was determined by the Kjeldahl method, and their protein contents (%) were obtained by multiplying the total nitrogen contents by the factor of 6.38 (Kurt et al., 2003). Fat content was determined according to Gerber centrifuge method and expressed as g fat/100 g kefir (AOAC, 1998).

#### 2.2.3 Color analyses

Color analyses were carried out by using a colorimeter device (Model CR‐400, Konica Minolta, Kyoto, Japan) in CIE (Commission International de L'Eclairage) LAB color system on the 1st, 4th, and 8th days of storage. Color measurements were taken by using reflectance specular included with D65 illuminant, 10° observer angle, and 8‐mm aperture. Each sample (5 mL) was placed in an optical glass cell provided by the manufacturer of the colorimeter (diameter of the cell 34 mm) and four measurements were taken at 3‐second intervals at approximately 5°C (Gursoy et al., 2016).

#### 2.2.4 Determination of total antioxidant activity and phenolic content

Preparation of extracts as follows. In the preparation of extracts, kefir samples were mixed and homogenized, then transferred into Eppendorf® tubes. The tubes were then centrifuged for 20 min at 12,225 × *g* by a microcentrifuge (WiseSpin, CF‐10, Daihan Scientific Co. Ltd., Gang‐Won‐Do, Korea). Total antioxidant activity and phenolic content analyses were performed by using supernatants obtained after centrifugation. Trolox®, diammonium salt of ABTS, Folin–Ciocalteu reagent, and gallic acid were purchased from Sigma‐Aldrich (St. Louis, MO, USA) while sodium carbonate was obtained from Riedel‐de Haen (Seelze, Germany).

The TEAC (Trolox® Equivalent Antioxidant Capacity) method was used for the determination of antioxidant activity (Cemeroğlu, 2007). For this purpose, the ammonium salt of ABTS (7 mM in water) was mixed with potassium persulfate (K_2_S_2_O_8_) (2.45 mM in water) by a ratio of 1:1 (v/v). To obtain ABTS stock solution, this solution was kept in dark at room temperature for 12–16 h. A linear calibration curve was obtained with Trolox® solution, and absorbance values were determined by a spectrophotometer (Optizen Pop, Mecasys Co., Ltd., Daejeon, Korea) at 724 nm. This stock solution was then diluted in chromatographic grade methanol to obtain a final absorbance value of 1.10 ± 0.02 at 734 nm to obtain the working solution of ABTS. During analyses, supernatant (150 μL) and ABTS working solution (2850 μL) were added into test tubes and mixed well by a vortexer (ViseMix, WM‐10, Daihan Scientific Co. Ltd., Gang‐Won‐Do, Korea), and this mixture was kept in dark for 30 min. Then, absorbance values were determined at 734 nm, and results are expressed as μmol Trolox® Equivalent (TE)/100 mL kefir.

Folin–Ciocalteu (FC) method was used to determine the total phenolic content of kefir samples (Cemeroğlu, 2007). During analyses, supernatant (0.5 mL) was transferred into a test tube, and FC reagent, which was previously diluted with distilled water (1:10, v/v) (2.5 mL), was added. After 3–5 min, 2 mL of 20% Na_2_CO_3_ (75 g/L) was added, and tubes were kept in dark for 2 h. Then, the absorbance values of samples were determined by a spectrophotometer at 760 nm, and results were expressed as mg gallic acid equivalent (GAE)/100 mL kefir.

#### 2.2.5 Determination of rheological properties

Rheological analyses were carried out at +4.0 ± 0.2°C by a viscometer (Model DV2T, Brookfield Engineering Laboratories, Middleboro, MA, USA) with an SC4‐21 spindle and a small sample adapter. The constant temperature was provided by a circulating water bath (Maxircu CR‐12, Daihan Scientific Co. Ltd., Gang‐Won‐Do, Korea), and the apparent viscosity values of samples were determined at 120 rpm (111.6 s^−1^). Flow behavior index (*n*) and consistency coefficient (*K*, Pa.s^*n*^) were calculated according to the power law model (Steffe, 1996).

#### 2.2.6 Microbiological analyses

*Lactobacillus* spp., *Lactococcus* spp., and yeast/mold counts of samples were determined on the 1st, 4th, and 8th days of storage. For microbiological analyses, kefir sample (10 mL) was mixed with sterile peptone water (90 mL) in a stomacher bag and homogenized by a stomacher (Bagmixer® 400P, Interscience, Saint‐Nom‐la‐Bretèche, France) for 2 min, and appropriate dilutions were prepared. *Lactobacillus* spp. was enumerated on MRS agar (Merck KGaA, Darmstadt, Germany) following 48 h incubation at 37 ± 1°C under anaerobic conditions while the number of *Streptococcus* spp. was enumerated on M17 agar (Merck KGaA, Darmstadt, Germany) following 48 h incubation at 37 ± 1°C under aerobic conditions (Colakoglu & Gursoy, 2011). Yeast/mold counts were enumerated on potato dextrose agar (PDA) (Merck KGaA, Darmstadt, Germany) acidified with L(+) tartaric lactic (10%, w/v) following 3–5 days at 25 ± 1°C (Kök‐Taş et al., 2013).

#### 2.2.7 Sensory analyses

Hedonic scales are extensively used to measure product liking and preference. The 9‐point hedonic scale may be the most informative sensory method used with a considerable success because naive consumers can understand the scale easily with minimal instruction, and results of this scale are “remarkably stable and product differences (in liking) are reproducible with different groups of subjects” (Stone & Sidel, 1992). However, our previous experience indicated that 9‐point scales might be nondistinctive and/or inappropriate for all native languages and subject segments as in the use of 7‐point face scales to measure children's responses to products. For this reason, sensory analyses in the present study were carried out by experienced panelists (20) on each storage day for measuring color, odor, taste, and overall likings with a 7‐point hedonic scale (1 = dislike extremely and 7 = like extremely) (Bodyfelt et al., 1988). All samples were numbered with three‐digit random codes and presented to each panelist in a different order. Panelists were instructed to drink water (Nazlı, Aydın, Turkey) between each sample to clean their palate.

#### 2.2.8 Statistical analysis

The analysis of variance (two‐way ANOVA) and the Duncan multiple‐comparison test were used at a significance level of α = 0.05 by means of the SAS package program (SAS System for Windows 9.0, SAS Institute Inc., Carry, NC, USA). Results were presented as mean ± standard deviation.

## 3 RESULTS AND DISCUSSION

### 3.1 Physicochemical properties

Dry matter, fat, and protein contents of kefir drinks on the first day of storage are presented in Table 1. On average, the dry matter contents of kefir drinks with PE at different ratios were between 16.91 and 17.20% while their fat contents ranged from 2.60% to 2.67%. Kefir drinks had a protein content in the range 2.80%–3.26%. According to Table 1, the addition of PE into kefir drinks at different ratios did not change their dry matter, fat, and protein contents significantly (*p* > .05). Moreover, the dry matter contents of plain kefir drinks without any table sugar were between 11.93 and 12.17%. Similarly, Wszolek et al. (2001) reported the mean dry matter content of kefir samples produced by using cow's milk as 11.67%. In the study by Gül (2017), the dry matter contents of kefir produced by adding 0%–4% mint juice were reported between 10.17% and 10.85%, and the differences in the dry matter contents of samples containing different ratios of mint juice were found insignificant. The dry matter contents of six different commercial kefir samples obtained from national markets in Turkey were in the range 9.49%–11.97% (Gursoy et al., 2020). In a study by Dinç (2008), the dry matter contents of fruit kefir drinks (*n* = 40) sold in the national markets of Ankara (Turkey) were found between 16.53% and 19.52%. Dry matter contents of both plain and strawberry‐flavored kefir samples in our study were in good agreement with the data reported in the literature.

**TABLE 1:** Dry matter, fat, and protein contents of kefir drinks with propolis extract.

Sample code^a^	Dry matter content^b^ (%)	Fat (%)	Fat (%, dry basis)	Protein (%)	Protein (%, dry basis)
A	16.91 ± 0.40^A^	2.65 ± 0.19^A^	15.98 ± 0.16^A^	2.80 ± 0.02^A^	16.59 ± 0.49^A^
B	17.01 ± 0.38^A^	2.62 ± 0.22^A^	15.84 ± 1.28^A^	3.02 ± 0.06^A^	17.72 ± 0.82^A^
C	17.20 ± 0.32^A^	2.67 ± 0.17^A^	15.82 ± 1.12^A^	3.01 ± 0.11^A^	17.66 ± 0.35^A^
D	17.17 ± 0.58^A^	2.60 ± 0.20^A^	15.64 ± 0.86^A^	3.26 ± 0.56^A^	19.07 ± 0.80^A^

^a^ A: Control, B: Kefir drink with 0.150% propolis extract, C: Kefir drink with 0.225% of propolis extract, and D: Kefir drink with 0.300% of propolis extract.

^b^ Different superscripts within the same column indicate that means are significantly different (*p* < .05).

Fat and protein contents of kefir drinks are highly dependent on the type of milk used in kefir production. Gursoy et al. (2020) reported the fat contents of six different commercial kefir drinks between 2.50% and 3.00% while their protein contents ranged from 2.30% to 3.44%. Öksüztepe et al. (2020) reported the average fat content of 2.13% in fruit kefir drinks, and the fat contents of kefir drinks with mint juice at varying ratios (0%–4%) were between 2.82% and 2.87% (Gül, 2017). Demir (2020) reported the protein contents of kefir drinks with rosehip marmalade (0%–15%) between 1.96% and 2.82% while Dinkçi et al. (2015) found the protein contents of kefir samples produced from a mixture of cow/oat milk between 1.53% and 3.48%. According to Turkish Fermented Dairy Products Communiqué (Turkish Food Codex (TFC), 2009), the milk fat content in kefir drinks should be less than 10%, and milk protein content of kefir and fermented milk products should be at least 2.7% on a weight basis. The protein and fat contents of kefir samples produced in this study were in good agreement with the national codex limits and literature data.

The pH and titratable acidity values of kefir drinks with PE at different ratios during storage are given in Table 2. The pH values of kefir drinks were between 3.96 and 4.10 while their titratable acidity values ranged from 0.74% and 0.83% during storage. The addition of PE did not influence the pH and acidity (lactic acid) values of kefir samples significantly (*p* > .05) but the pH values of kefir drinks decreased (*p* < .05) while their acidity values increased during storage (*p* < .05). Similar to our study, using different kefir grains, Garrote et al. (2001) reported the pH values of kefir drinks between 3.69 and 3.83. Moreover, Chon et al. (2020) reported the pH values of kefir samples with different ratios of propolis (0%–2%) in the range 3.70–3.75, and differences in pH values were found insignificant among kefir drinks with different propolis ratios. Gursoy et al. (2020) reported that commercial kefir drinks sold in national markets had a pH value between 3.86 and 4.06 while their titratable acidity (%lactic acid) values ranged from 0.71% to 0.93% in Turkey. In a study by Ünlütürk and Turantaş (1998), the lactic acid contents of kefir samples were found between 0.9% and 1.1% for different fermentation times. In the Turkish Fermented Dairy Products Communiqué (Turkish Food Codex (TFC), 2009), the titratable acidity of kefir drinks should be at least 0.6%. The titratable acidity values of kefir drinks produced in the current study were in good agreement with the national codex limit.

**TABLE 2:** Changes in the pH, titratable acidity, and color parameters of kefir drinks with propolis extract during storage.

Sample code^a^	Storage time (day)	pH^b^	Titratable acidity (% lactic acid)	L*	a*	b*
A	1	4.10 ± 0.09^A^	0.75 ± 0.01^E^	84.89 ± 0.03^A^	−2.47 ± 0.02^A^	5.37 ± 0.20^CD^
	4	3.99 ± 0.03^CD^	0.77 ± 0.02^CDE^	85.06 ± 0.62^A^	−2.52 ± 0.15^AB^	5.45 ± 0.75^CD^
	8	3.96 ± 0.05^D^	0.81 ± 0.012^ABC^	84.75 ± 0.34^A^	−2.62 ± 0.23^ABC^	5.26 ± 0.69^D^
B	1	4.07 ± 0.20^A^	0.74 ± 0.009^E^	84.53 ± 0.06^A^	−2.57 ± 0.03^AB^	6.24 ± 0.09^ABC^
	4	4.04 ± 0.02^B^	0.76 ± 0.02^DE^	84.71 ± 0.66^A^	−2.63 ± 0.2^ABC^	6.42 ± 0.51^AB^
	8	3.96 ± 0.03^D^	0.81 ± 0.06^ABC^	85.03 ± 1.18^A^	−2.63 ± 0.36^ABC^	5.53 ± 1.45^BCD^
C	1	4.10 ± 0.008^A^	0.78 ± 0.038^CDE^	84.72 ± 0.17^A^	−2.63 ± 0.06^ABC^	6.74 ± 0.09^A^
	4	4.01 ± 0.03^BC^	0.81 ± 0.01^ABC^	84.93 ± 0.31^A^	−2.74 ± 0.82^BC^	6.88 ± 0.53^A^
	8	3.97 ± 0.01^D^	0.83 ± 0.02^A^	84.50 ± 0.36^A^	−2.82 ± 0.23^C^	6.68 ± 0.58^A^
D	1	4.10 ± 0.012^A^	0.76 ± 0.02^DE^	84.56 ± 0.17^A^	−2.69 ± 0.02^ABC^	6.95 ± 0.05^A^
	4	4.02 ± 0.230^BC^	0.79 ± 0.01^BCD^	84.45 ± 0.63^A^	−2.69 ± 0.08^ABC^	7.12 ± 0.49^A^
	8	3.97 ± 0.05^D^	0.82 ± 0.01^AB^	85.50 ± 2.23^A^	−2.83 ± 0.15^C^	6.95 ± 0.72^A^

^a^ A: Control, B: Kefir drink with 0.150% propolis extract, C: Kefir drink with 0.225% of propolis extract, and D: Kefir drink with 0.300% of propolis extract.

^b^ Different superscripts within the same column indicate that means are significantly different (*p* < .05).

### 3.2 Color parameters

The color parameters of kefir drinks with PE at different ratios during storage are given in Table 2. The addition of PE did not have a significant effect on the L* color values of strawberry‐flavored kefir drinks (*p* > .05). During storage, the L* color values of kefir samples varied between 84.45 and 85.50, and the interaction of PE ratio with storage time on the L* color values of kefir drinks was also found insignificant (*p* > .05). The individual effect of PE addition on the a* color values of kefir drinks was insignificant (*p* > .05). On the other hand, the addition of PE into kefir drinks increased the b* (yellowness) color values of samples significantly (*p* < .05), and this value varied between 5.37 and 7.12 during storage. It is considered that this situation is caused by the yellowish color of PE. Additionally, it was found that the individual effect of storage time on the L*, a*, and b* color values of kefir samples was insignificant (*p* > .05). Adding propolis to yogurt samples at different ratios (0.25%–0.75%), Çifci (2015) reported that Hunter L and color values of yogurt samples decreased and b color values increased as storage time and the propolis ratio increased.

### 3.3 Rheological properties

Rheological properties of kefir drinks with PE at different ratios during storage are given in Table 3. On average, the apparent viscosity values of kefir drinks were between 145.52 and 194.60 mPa.s, and neither the addition of PE nor storage time had a statistically significant effect on the rheological properties of kefir samples (*p* > .05). The consistency coefficients (*K*) of kefir samples varied between 4236.75 and 20,302.00 mPa.s^*n*^ while their flow behavior index (*n*) was in the range 0.03–0.34. All kefir samples showed a pseudoplastic flow behavior (i.e., *n* < 1). The highest *n* value (0.34) was determined on the 1st day of storage for sample D while the lowest (0.03) was found on the 1st day of storage for sample B. The difference in *n* values among samples was found statistically significant (*p* < .05).

**TABLE 3:** Changes in the rheological properties of kefir drinks with propolis extract during storage.

Kefir sample^a^	Storage time (day)	Apparent viscosity^b^ (mPa.s, at 111.6 s^−1^)	Consistency coefficient (*K*, mPa.s^*n*^)	Flow behavior index (*n*)	Determination coefficient (*R* ^2^)
A	1	194.60 ± 28.57^A^	17,250.75 ± 18,104.59^AB^	0.14 ± 0.25^AB^	0.98 ± 0.014
	4	172.32 ± 21.03^ABCD^	8814.00 ± 2559.50^AB^	0.17 ± 0.08^AB^	0.99 ± 0.00
	8	148.45 ± 3.04^CD^	4236.75 ± 3059.63^B^	0.26 ± 0.08^AB^	0.76 ± 0.45
B	1	171.20 ± 25.37^ABCD^	20,302.00 ± 6327.80^A^	0.03 ± 0.07^B^	0.98 ± 0.004
	4	148.32 ± 11.56^CD^	8284.00 ± 4580.39^AB^	0.17 ± 0.13^AB^	0.98 ± 0.009
	8	145.52 ± 18.33^D^	4684.25 ± 2127.54^B^	0.29 ± 0.16^AB^	0.99 ± 0.002
C	1	180.62 ± 27.82^ABC^	15,710.25 ± 13,688.27^AB^	0.14 ± 0.23^AB^	0.98 ± 0.009
	4	182.20 ± 19.39^AB^	12,422.75 ± 13,621.14^AB^	0.08 ± 0.17^AB^	0.98 ± 0.007
	8	163.43 ± 11.82^ABCD^	7207.0 ± 5743.1502^AB^	0.24 ± 0.13^AB^	0.99 ± 0.002
D	1	163.45 ± 43.72^ABCD^	10,559.50 ± 9673.72^AB^	0.34 ± 0.41^A^	0.98 ± 0.01
	4	150.05 ± 28.47^BCD^	8371.00 ± 5464.34^AB^	0.19 ± 0.07^AB^	0.99 ± 0.02
	8	164.12 ± 10.06^ABCD^	5966.50 ± 2271.15^B^	0.26 ± 0.11^AB^	0.99 ± 0.03

^a^ A: Control, B: Kefir drink with 0.150% propolis extract, C: Kefir drink with 0.225% of propolis extract, and D: Kefir drink with 0.300% of propolis extract.

^b^ Different superscripts within the same column indicate that means are significantly different (*p* < .05).

**FIGURE 1:**
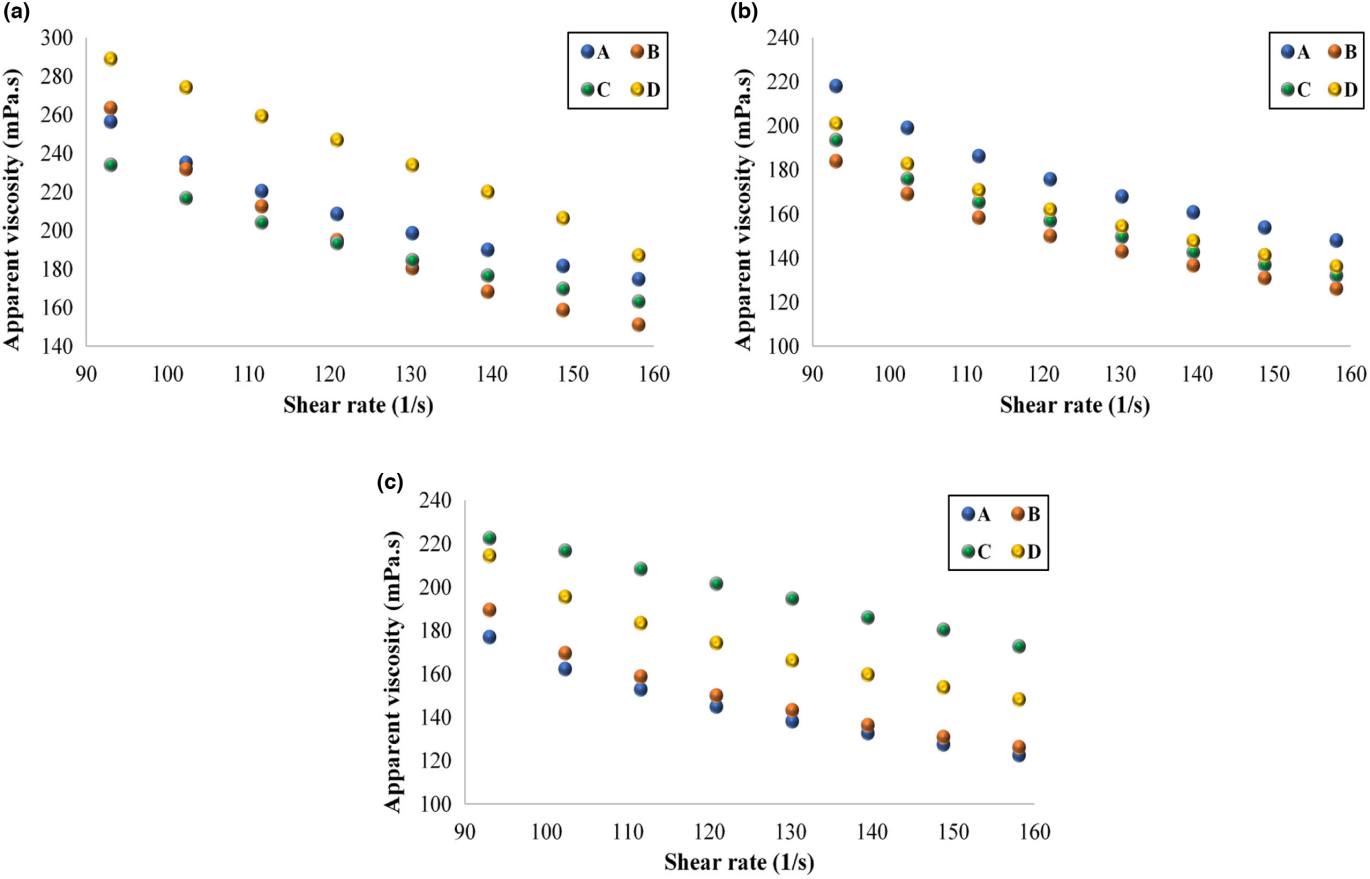
The flow behavior of kefir drinks with propolis extract on the 1st day (a), the 4th day (b), and the 8th day of storage at 4°C (c) (A: Control, B: Kefir drink with 0.150% propolis extract, C: Kefir drink with 0.225% of propolis extract, and D: Kefir drink with 0.300% of propolis extract).

The changes in the apparent viscosity values of kefir drinks with PE at different shear rates are shown at different storage days in Figure 1, and the apparent viscosities and consistency coefficients of kefir samples decreased with storage time (Table 3). Since the flow behavior indices between 0 < *n* < 1, kefir drinks with PE at different ratios exhibited a non‐Newtonian pseudoplastic flow behavior. Similarly, a variety of kefir drinks were reported to show a pseudoplastic flow behavior in the literature (Doğan, 2011; Gursoy et al., 2020; Kök‐Taş et al., 2013; Tratnik et al., 2006).

The mean apparent viscosity values of plain kefir samples produced from cow milk were reported as 101, 89, and 75 mPa.s by Tratnik et al. (2006) on the 1st, 5th, and 10th days of storage, respectively. The average viscosities of plain, fruit, and diet kefir drinks marketed in Ankara (Turkey) were determined as 104.4, 120, and 111 mPa.s, respectively (Uslu, 2010). Kök‐Taş et al. (2013) reported the apparent viscosities of kefir drinks with a fat content of 0.1% between 2.02 and 2.47 Pa.s when kefir drinks were stored for up to 21 days. In a study by Doğan (2011), different ratios of honey (10, 20, and 30, w/w) were added to kefir, and an increase in honey concentration decreased the consistency coefficients of kefir drinks. The apparent viscosities of kefir drinks at a shear rate of 50 s^−1^ were found between 15.39 and 16.35 mPa.s while all samples exhibited a pseudoplastic behavior. Irigoyen et al. (2005) studied the rheological properties of kefir with kefir grains at different ratios during storage (28 days) at 5 ± 1°C, and reported that their viscosity values decreased significantly during storage. In a study by Gursoy et al. (2020), the apparent viscosity values of commercial kefir samples (4, 10, and 25°C) were between 0.48 and 1.76 mPa (111.6 s^−1^) at three different temperatures while their flow behavior indices varied from 0.18 to 0.44, indicating a pseudoplastic flow behavior. The results of the present study were in good agreement with the data reported in the literature.

### 3.4 Total phenolic content and antioxidant activity

The phenolic content and antioxidant activity values of kefir drinks with PE at different ratios during storage at 4°C are shown in Table 4. The total antioxidant capacity of kefir samples (100 mL) was between 1.97 and 2.51 μmol TE while their total phenolic content (100 mL) ranged from 11.55 to 13.98 mg GAE. The total antioxidant capacity and phenolic content of kefir samples increased with an increase in the ratio of PE, and they decreased slightly during storage. While the lowest total antioxidant capacity was found for the kefir samples A and B on the 8th day of storage, it was 2.51 μmol TE/100 mL for the kefir sample D on the 1st day of storage. In general, changes in the total phenolic contents of kefir samples were in parallel with their total antioxidant activity values.

**TABLE 4:** Changes in the total phenolic content and antioxidant activity values of kefir drinks with propolis extract during storage.

Sample code^a^	Storage time (day)	Total phenolic content^b^ (mg GAE/100 mL)	Total antioxidant activity (μmol TE/100 mL)
A	1	12.53 ± 1.41^ABCD^	2.19 ± 0.20^D^
	4	11.85 ± 0.85^CD^	2.21 ± 0.17^D^
	8	11.55 ± 1.30^D^	1.97 ± 0.14^E^
B	1	13.39 ± 1.54^ABCD^	2.35 ± 0.082^BC^
	4	12.74 ± 1.49^ABCD^	2.29 ± 0.12^CD^
	8	12.05 ± 1.52^BCD^	2.04 ± 0.063^E^
C	1	13.87 ± 1.80^AB^	2.44 ± 0.054^AB^
	4	13.79 ± 1.64^ABC^	2.46 ± 0.040^AB^
	8	12.98 ± 2.31^ABCD^	2.19 ± 0.026^D^
D	1	14.07 ± 2.03^A^	2.51 ± 0.046^A^
	4	13.98 ± 1.65^AB^	2.46 ± 0.029^AB^
	8	13.14 ± 2.36^ABCD^	2.20 ± 0.022^D^

^a^ A: Control, B: Kefir drink with 0.150% propolis extract, C: Kefir drink with 0.225% of propolis extract, and D: Kefir drink with 0.300% of propolis extract.

^b^ Different superscripts within the same column indicate that means are significantly different (*p* < .05).

Adding different ratios of raw propolis powder into ayran (0%–0.75%, w/v) and buttermilk (1%–3%, w/v), Çelik (2016) reported that increasing the ratio of propolis powder in both products resulted in an increase in their DPPH radical scavenging activities and phenolic contents, which decreased during storage. Mehmetoğlu (2019) reported that the addition of propolis (0.1%–0.5%) into ice creams significantly increased their antioxidant activities. Aktaş (2019) used microencapsulated PE in the production of banana‐flavored pudding and stated that the antioxidant activities and phenolic contents of pudding samples significantly increased with an increase in the ratio of propolis while there was a decrease in these values during storage. Santos et al. (2019) produced probiotic yogurt samples by using PE (0.05%, w/v), pasteurized strawberry pulp (10%, w/v), and sugar (12.6%) and determined their total phenolic contents between 5.49 and 5.73 mg GAE/g, which decreased during 28 days of storage. Studying the potential use of propolis as an alternative to food additives like sodium benzoate and potassium sorbate in the preservation of orange juice, Yang et al. (2017) reported that the use of propolis could effectively protect the antioxidant capacity of orange juice. The results of this present study were in good agreement with literature data.

### 3.5 Microbiological properties

*Lactobacillus* spp., *Lactococcus* spp., and yeast–mold counts of kefir drinks with PE at different ratios during storage at 4°C are shown in Table 5. The ratio of PE did not have a statistically significant effect on the total *Lactobacillus* spp., *Lactococcus* spp., and yeast counts (log CFU/mL) of kefir drinks (*p* > .05). According to the Turkish Food Codex Fermented Dairy Products Communiqué (Turkish Food Codex (TFC), 2009), all strawberry‐flavored kefir drinks complied with the microbiological quality criteria that are specified. In this Communiqué, it is reported that at least 7 log of total specific microorganisms (CFU/g) and at least 4 log of yeasts (CFU/g) must be present in kefir drinks. Therefore, the microbial content and stability are the most important quality factors of kefir drinks. The microbial content of kefir drinks produced in the current study was in accordance with these criteria.

**TABLE 5:** Changes in the total *Lactobacillus* spp., *Lactococcus* spp., and yeast–mold counts (log CFU/mL) of kefir drinks with propolis extract during storage.

Sample code^a^	Storage time (day)	*Lactobacillus* spp.^b^	*Lactococcus* spp.	Mold‐yeast
A	1	6.40 ± 0.07^A^	8.40 ± 0.02^A^	4.30 ± 0.01^A^
	4	6.22 ± 0.07^A^	8.28 ± 0.04^A^	4.35 ± 0.00^A^
	8	6.17 ± 0.12^A^	8.21 ± 0.19^A^	4.37 ± 0.00^A^
B	1	6.29 ± 0.06^A^	8.37 ± 0.01^A^	4.25 ± 0.00^A^
	4	6.14 ± 0.10^A^	8.21 ± 0.07^A^	4.27 ± 0.01^A^
	8	6.03 ± 0.00^A^	8.12 ± 0.14^A^	4.31 ± 0.00^A^
C	1	6.18 ± 0.02^A^	8.26 ± 0.02^A^	4.19 ± 0.00^A^
	4	6.10 ± 0.09^A^	8.12 ± 0.06^A^	4.21 ± 0.00^A^
	8	6.07 ± 0.05^A^	8.05 ± 0.07^A^	4.24 ± 0.00^A^
D	1	6.14 ± 0.02^A^	8.19 ± 0.04^A^	4.12 ± 0.06^A^
	4	6.05 ± 0.06^A^	8.04 ± 0.02^A^	4.13 ± 0.03^A^
	8	5.95 ± 0.06^A^	7.97 ± 0.02^A^	4.16 ± 0.02^A^

^a^ A: Control, B: Kefir drink with 0.150% propolis extract, C: Kefir drink with 0.225% of propolis extract, and D: Kefir drink with 0.300% of propolis extract.

^b^ Different superscripts within the same column indicate that means are significantly different (*p* < .05).

Kök‐Taş et al. (2013) determined the number of *Lactobacillus* spp. In kefir drinks produced from cow's milk as 9.27 log CFU/mL on the 1st day of storage, and this value decreased during 21 days of storage. Çifci (2015) reported that the use of 0.25%, 0.50%, and 0.75% propolis and 0.50% honey in the production of yogurt samples did not significantly influence the *Streptococcus salivarus* subsp. *thermophilus* numbers; however, *L. delbrueckii* subsp. *bulgaricus* and yeast–mold numbers decreased significantly during storage. Additionally, the author reported that the number of yeasts–molds significantly reduced in yogurt samples by the addition of propolis. In kefir drinks produced by using powdered milk and different ratios of fruit sauces, Ak (2018) reported that the total number of lactic acid bacteria decreased with an increase in storage time. In our study, unlike literature data, there was no decrease in the number of microorganisms in kefir drinks during storage. This difference might be due to the properties of PE used, its ratio in kefir drinks, and storage time. The insignificant increase in the number of yeasts–molds in kefir samples during storage might be explained by a decrease in pH and the presence of more suitable conditions for yeast and mold growth.

### 3.6 Sensory properties

**FIGURE 2:**
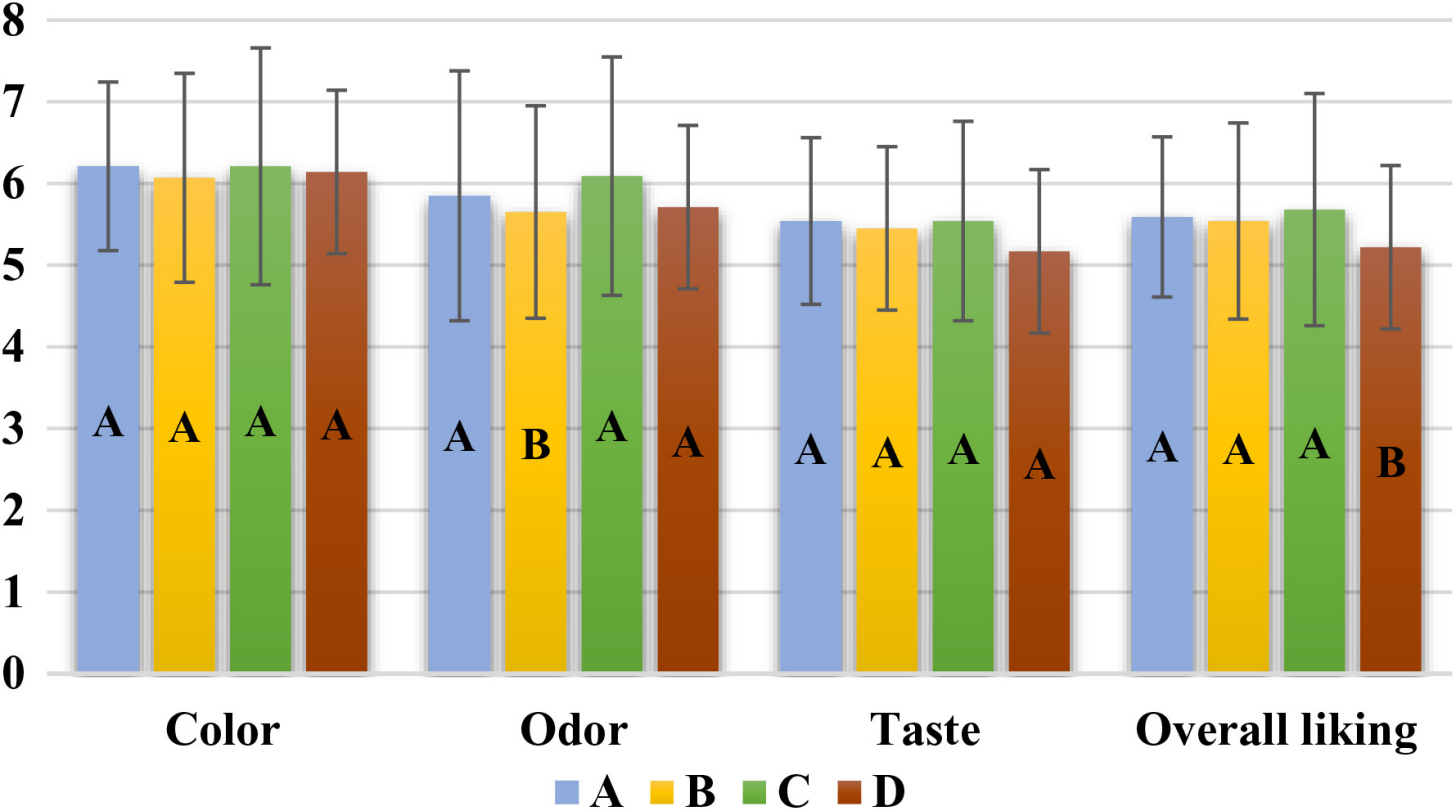
The individual effect of propolis extract ratio on the sensory properties of kefir drinks when the effect of storage time was averaged out (different letters within each sensory parameter bar in the figure indicate that means are significantly different (*p* < .05). (A: Control, B: Kefir drink with 0.150% propolis extract, C: Kefir drink with 0.225% propolis extract, and D: Kefir drink with 0.300% propolis extract).

The sensory properties of kefir drinks with PE at different ratios during storage at 4°C are shown in Table 6 and Figure 2. On the 1st and 4th days of storage, all kefir drinks received similar scores in terms of color, odor, taste, and overall liking (*p* > .05). For all sample groups, all sensory scores decreased significantly for the 8th day of storage compared to the 1st day (*p* < .05) (Table 6). The addition of PE did not result in any adverse effects on color‐ and taste‐liking scores of kefir samples (Figure 2) (*p* > .05). Kefir samples C and D had an odor‐liking score similar to the control kefir drink (A), which also had an overall liking score similar to kefir samples B and C with the PE at a ratio of 0.225%. During storage, the color, odor, taste, and overall liking scores of all kefir samples decreased, and this decrease was found statistically significant on the 8th day of storage (*p* < .05). In terms of all sensory parameters, even though kefir drinks A and C had the highest value, differences in sensory scores among kefir samples were statistically insignificant (*p* > .05) (Figure 2).

**TABLE 6:** Changes in the sensory properties of kefir drinks with propolis extract during storage.

Sample code^a^	Storage time (day)	Color^b^	Odor	Taste	Overall liking
A	1	6.50 ± 0.81^A^	6.17 ± 1.17^AB^	5.97 ± 0.99^A^	5.85 ± 1.05^AB^
	4	6.50 ± 0.71^A^	5.95 ± 1.37^B^	5.92 ± 1.24^A^	5.8 ± 1.29^AB^
	8	5.65 ± 1.21^CD^	5.42 ± 1.21^CD^	4.72 ± 1.69^CD^	5.07 ± 1.34^DE^
B	1	6.45 ± 0.67^A^	5.97 ± 1.09^AB^	5.90 ± 1.23^A^	6.05 ± 0.98^A^
	4	6.30 ± 0.96^AB^	6.15 ± 1.01^AB^	5.92 ± 1.11^A^	5.95 ± 1.03^AB^
	8	5.47 ± 1.44^D^	4.82 ± 1.37^E^	4.55 ± 1.56^D^	4.62 ± 1.27^EF^
C	1	6.32 ± 1.09^AB^	6.45 ± 0.55^A^	5.90 ± 1.21^A^	6.02 ± 1.02^A^
	4	6.40 ± 0.81^A^	6.07 ± 1.14^AB^	5.57 ± 1.12^AB^	5.80 ± 0.85^AB^
	8	5.92 ± 1.09^BC^	5.75 ± 1.10^BC^	5.15 ± 1.25^BC^	5.22 ± 0.97^CD^
D	1	6.35 ± 0.69^AB^	6.15 ± 0.76^AB^	5.55 ± 1.29^AB^	5.72 ± 1.26^ABC^
	4	6.30 ± 0.79^AB^	6.02 ± 1.07^AB^	5.42 ± 1.15^AB^	5.45 ± 1.23^BCD^
	8	5.77 ± 1.29^CD^	4.97 ± 1.34^DE^	4.55 ± 1.61^D^	4.50 ± 1.56^F^

^a^ A: Control, B: Kefir drink with 0.150% propolis extract, C: Kefir drink with 0.225% of propolis extract, and D: Kefir drink with 0.300% of propolis extract.

^b^ Different superscripts within the same column indicate that means are significantly different (*p* < .05).

Chon et al. (2020) added propolis into plain kefir drinks at different ratios (0%–2%) and reported that the sensory scores of kefir samples decreased significantly compared to the control group as the ratio of propolis increased. Determining the sensory properties of yogurts with propolis and honey at different ratios during storage, Çifci (2015) reported that the odor and texture scores of samples decreased as propolis ratio and storage time increased. Mehmetoğlu (2019) used propolis powder (0.1%–0.5%) in ice cream production and reported its adverse effect on the sensory properties of ice cream samples while the most liked one was the control sample. Adding microencapsulated PE into cake samples at different ratios (2.5%, 5.0%, 7.5%, and 10.0%, w/w), Acun (2021) found that cakes with more than 5.0% PE were not preferred by panelists. Results in the present study were generally in good agreement with the literature. Since the ratio of PE added to strawberry‐flavored kefir drinks was relatively low in comparison to previous studies, their sensory liking scores were similar to those of the control drinks.

## 4 CONCLUSION

The addition of PE into kefir drinks and storage time of up to 8 days at 4°C did not have a significant effect on the dry matter, protein, and fat contents of drinks. The pH values of drinks decreased while their titratable acidity values increased with an increase in storage time. The addition of PE into strawberry‐flavored kefir drinks and storage time did not have a significant effect on their L* and a* color values, but b* color values increased as the ratio of PE increased. Generally, the addition of PE and storage time did not change the rheological properties of kefir samples, which showed a pseudoplastic flow behavior. It was found that the total antioxidant capacity and phenolic content of kefir drinks increased with an increase in the ratio of PE while these values decreased during storage. The addition of PE did not influence the sensory color and taste liking scores of kefir drinks, and the overall liking scores of kefir drinks with PE at a ratio of up to 0.225% were similar to those of the control drinks. The addition of PE had an insignificant effect on the total *Lactobacillus* spp., *Lactococcus* spp., and yeast counts of kefir drinks. Results indicated that it was possible to produce a strawberry‐flavored kefir drink with 0.225% PE, which could be acceptable in terms of its sensory and increased functional properties, and it could be stored at 4°C for up to 8 days. This functional kefir drink can be consumed favorably by a wide range of populations, especially children.

## AUTHOR CONTRIBUTIONS

**Sinem Bengi:** Conceptualization (equal); formal analysis (lead); investigation (lead); methodology (equal); writing – original draft (equal). **Oguz Gursoy:** Conceptualization (lead); investigation (lead); methodology (lead); resources (lead); supervision (lead); validation (lead); writing – review and editing (lead). **Hande Özge Güler Dal:** Conceptualization (equal); formal analysis (supporting); investigation (supporting); writing – original draft (equal). **Yusuf Yilmaz:** Conceptualization (equal); formal analysis (supporting); investigation (supporting); methodology (supporting); writing – review and editing (equal).

## CONFLICT OF INTEREST STATEMENT

The authors confirm that they have no conflicts of interest with respect to the work described in this manuscript.

## Data Availability

Data will be made available on request.
